# Impact of sperm DNA chromatin in the clinic

**DOI:** 10.1007/s10815-015-0624-x

**Published:** 2015-12-17

**Authors:** Dimitrios Ioannou, David Miller, Darren K. Griffin, Helen G. Tempest

**Affiliations:** Department of Human and Molecular Genetics, Herbert Wertheim College of Medicine, Florida International University, Miami, FL USA; Leeds Institute of Genetics, Health and Therapeutics, University of Leeds, Leeds, UK; School of Biosciences, University of Kent, Canterbury, CT2 7NJ UK; Biomolecular Sciences Institute Florida International University, Miami, FL USA

**Keywords:** Chromatin, Genetic integrity, Aneuploidy, Sperm DNA fragmentation

## Abstract

The paternal contribution to fertilization and embryogenesis is frequently overlooked as the spermatozoon is often considered to be a silent vessel whose only function is to safely deliver the paternal genome to the maternal oocyte. In this article, we hope to demonstrate that this perception is far from the truth. Typically, infertile men have been unable to conceive naturally (or through regular IVF), and therefore, a perturbation of the genetic integrity of sperm heads in infertile males has been under-considered. The advent of intracytoplasmic sperm injection (ICSI) however has led to very successful treatment of male factor infertility and subsequent widespread use in IVF clinics worldwide. Until recently, little concern has been raised about the genetic quality of sperm in ICSI patients or the impact genetic aberrations could have on fertility and embryogenesis. This review highlights the importance of chromatin packaging in the sperm nucleus as essential for the establishment and maintenance of a viable pregnancy.

## Introduction

Currently, there are limited diagnostic tests available to evaluate the genetic integrity of a semen sample as, traditionally, male fertility evaluation has used conventional semen parameters (concentration, motility morphology, etc.) Although providing fundamental information, it has been strongly suggested that this basic evaluation has questionable clinical value [1]. Within recent years, therefore, several molecular genetic techniques have been developed to assess the genetic integrity of sperm. This review will focus on our current understanding of the genetic integrity of sperm and its impact on fertility and embryogenesis.

The spermatozoon is a highly elaborate and specialized cell that is formed through the process of spermatogenesis, during which a complex cellular program of differentiation occurs. The end result is mature spermatozoa that are essential for reproduction, fertilization, and normal embryo development. The sperm cell is unique in morphology, chromatin structure, and function, and the process of spermatogenesis is characterized by a myriad of changes. Essentially, these can be broken down into three sequential elements: (i) mitotic proliferation (producing large numbers of spermatocytes); (ii) meiotic recombination and chromosome segregation (producing genetically diverse haploid gametes); and (iii) culminates in cytodifferentiation (re-packaging of the haploid genome for delivery to the oocyte). In general, transcription and translation are temporarily disengaged during the post-meiotic stage of spermatogenesis (when round cells are extensively remodeled to form mature spermatozoa) [2]. Furthermore, the storage of mRNAs encoding for protamines is crucial for the completion of spermiogenesis [3]. The structure of sperm chromatin and perturbations in its integrity that could have a clinical impact in the form of chromosome aneuploidy, DNA fragmentation, or chromatin organization will form the backbone of this review. This review thus addresses the impact of the unique features of the genetic integrity of sperm in a clinical setting.

### Structure of human sperm chromatin

DNA is, in somatic nuclei complexed, condensed and packaged with a family of proteins (histones) into nucleosomes (chromatin). Histones can be modified by post-translational modifications that regulate the degree of DNA compaction and modulate gene expression by restricting or facilitating access of transcription factors to the DNA. Despite the efficacy of histones in regulating DNA compaction and gene expression, it is clear that an alternative mechanism for DNA packaging has evolved in sperm cells. The haploid sperm chromatin undergoes one of the most significant changes known in biology, in which, the vast majority of histones are replaced with transition proteins, followed by protamines resulting in a highly condensed (typically 10 % or less volume than other somatic cell nucleus) [2], relatively transcriptionally inert cell [4, 5]. Normal human sperm chromatin remains packaged by 5–15 % of histones [6, 7], a higher percentage compared to other mammalian species, e.g., bulls, stallions, hamsters, and mice, which retain (<5 %) [8]. Therefore, compared to other mammalian species, human sperm chromatin is relatively less compact. Interestingly, dye-binding assays such as aniline blue staining that selectively stain histones are often used as markers of sperm immaturity [9, 10]. As discussed later, this has a bearing on recent reports suggesting an epigenetic role for retained histones (particularly histones carrying post-translational modifications) in sperm.

During chromatin remodeling/compaction in sperm, naturally occurring DNA strand breaks induced by topoisomerase II arise to relieve the torsional stresses that accompany the transition of sperm chromatin from an exclusively nucleosomal to a predominantly protamine-based configuration [11]. Re-packaging with protamines most likely evolved to more efficiently compact chromatin into as small a volume as possible and so facilitate safe delivery of the paternal DNA to the oocyte [2]. Arginine, which is the main component of protamines, is responsible for allowing a higher degree of chromatin compaction by neutralizing the strong negative charges of the phosphate groups in the DNA backbone [12]. Cysteine residues confer extra stability through intermolecular disulfide cross-links [13]. These allow the basic protamine packaging unit (toroid) to be further condensed, with each chromosome becoming a garland of toroids, clustered by centromeres that form a chromo-center in the nuclear interior. The telomeres on the other hand form dimers and tetramers at the nuclear periphery [14–17]. Evidence for regions of the sperm genome remaining associated with nucleosomes that retain a more open conformation comes from studies using salt-washing and/or limited endonuclease digestion of sperm nuclei [18–23]. In two such studies, nucleosome-rich, low salt-washed, and restriction (or micrococcal nuclease) digestion-sensitive regions were shown to be associated with important developmental gene sequences involved in embryogenesis [18, 22]. Other studies suggested that these regions appear to be associated with hypomethylated DNA [24, 25], indicating a potentially permissive state for transcriptional activation of specific gene families involved in early cell differentiation and embryogenesis, perhaps in the early embryo [7]. Altered patterns of sperm DNA and histone methylation have also been reported in infertile men [25], supporting earlier studies into the relationship between DNA methylation and pregnancy outcome in an IVF setting [26]. Additionally, in comparison to other mammalian species (e.g., bull cat, boar, ram) that contain one type of protamine (P1), mouse and human sperm contain two types of protamines (P1 and P2) [27], both present in roughly equal quantities. Improper temporal regulation of these transcripts leading to altered expression of the mature proteins is associated with male infertility especially when the 1:1 ratio is perturbed [27, 28]. Furthermore, an altered P1/P2 ratio has been correlated with a negative impact on embryo quality and IVF outcome although at least one study has shown that a fertility-defining 1:1 ratio is equivocal [29]. As indicated above, infertile men often present with an increased histone to protamine ratio compared to fertile counterparts with a subset of infertile men possessing complete protamine deficiency [30]. In this regard, mouse knockout models in which the intermediate DNA re-packaging transition proteins are lost show impaired fertility and protamine knock-outs are completely infertile [31, 32]. Therefore, the incorrect distribution of histones and protamines throughout the genome could have undesired effects on early embryo development [2, 27].

### Sperm aneuploidy and its impact in the clinic

Chromosome aneuploidy (the presence of extra or missing chromosomes in the sperm head) impacts on embryogenesis and the health and development of future offspring. Chromosome aneuploidy is clinically significant, given that it is the leading cause of pregnancy loss and mental retardation in humans. Embryonic aneuploidy can arise from several different mechanisms: (1) a non-disjunction event giving rise to an aneuploid sperm or oocyte, resulting in chromosomally unbalanced gametes or (2) a mitotic loss (e.g., through anaphase lag), gain, or non-disjunction event in the embryo, leading to mosaicism with the presence of normal and aneuploid cells [33]. The parental origin of aneuploidies has been reviewed extensively elsewhere [33–37]. In brief, autosomal trisomies and trisomy X are typically maternal in origin (70–100 %). Thus, aneuploidy is predominantly considered to be maternal in origin, with advanced maternal age being a significant risk factor for increased risk of aneuploidy [33]. However, it should be noted that a paternal contribution remains for the aforementioned trisomies. Additionally, certain clinically viable aneuploidies (e.g., monosomy X, 47, XXY,and 47, XYY) are predominantly paternal in origin with a prevalence of 70–80, 50, and 100 %, respectively [38]. Advancing paternal age has less impact on chromosome aneuploidy with only a slight increased risk emerging for certain chromosomes [39].

Sperm aneuploidy can be readily identified by either sperm karyotyping (achieved after fertilization of a human sperm with a hamster oocyte) or by interphase fluorescence in situ hybridization (FISH) in human sperm nuclei. Early sperm aneuploidy studies studied sperm karyotypes, a technically demanding and labor-intensive method that could only study a relatively low number of sperm cells from each individual, that were capable of fertilizing a hamster oocyte. The major benefit of this method is that it allows both numerical and structural chromosome aberrations to be identified. The advent of FISH revolutionized our ability to study sperm aneuploidy allowing thousands of sperm to be studied and was rapidly adopted as a research tool to investigate the paternal contribution to aneuploidy. Early cumulative data from the sperm karyotype studies revealed a higher incidence of structural chromosome compared to numerical aberrations within sperm cells (6–7 % versus 1–2 %, respectively) [34, 40]. However, this is based on a relatively small number of karyotypes and subjects. Therefore, for the remainder of this section, we will focus on FISH studies. Several comprehensive reviews have been published compiling previous studies evaluating sperm aneuploidy levels in fertile and infertile patient cohorts for all chromosomes [38, 41–43]. Taken together, the vast majority of studies have demonstrated that all men produce a proportion of aneuploid sperm. However, an increased percentage of sperm aneuploidy has been significantly associated with reduced semen parameters and shown to increase with the severity of the male factor infertility [38, 41–43]. Furthermore, specific chromosomes namely chromosomes 21, 22 and X, and Y typically display a two- to threefold increase in chromosome non-disjunction compared to the rest of the chromosome complement [38, 41–43]. This is perhaps not surprising as meiotic recombination plays a crucial role in keeping homologous chromosomes together. The aforementioned chromosomes have the smallest pairing regions and usually only present with single chiasmata [40, 44, 45].

The emerging sperm aneuploidy picture from studying males with different types of infertility (e.g., oligo-, astheno-, terato-, zoospermia) is that they have on average of threefold increase with a two- to tenfold higher prevalence of numerical chromosome abnormalities compared to their fertile counterparts [44, 46]. Patients with more severe morphological abnormalities in sperm (e.g., macrocephalic-multi-flagellated sperm, globozoospermia) manifest 10–30× and 8–10× increase in aneuploidy, respectively, compared to controls [38, 47, 48]. Patients with oligoasthenoteratozoospermia (OAT) have been studied extensively using FISH probes by many groups, and an increased level (up to 30-fold) of aneuploidies (disomy, diploidy, nullisomy) has been found for all investigated chromosomes compared to their fertile counterparts [42, 46, 47, 49–56].

In terms of sperm aneuploidy in individuals with numerical or structural chromosome aberrations (e.g., 47,XXY, 47,XYY, Robertsonian, or reciprocal translocations) it appears that the actual observed rates are lower from the theoretical numbers expected from the behavior of the meiotic trivalents or quadrivalents possibly due to some selection against these aneuploid sperm (e.g., an unknown meiotic checkpoint) [38, 47, 48, 56, 57]. Non-mosaic Klinefelter patients are reported to have an average of 6 % sex chromosome aneuploidy within their sperm [47]. The proportion of unbalanced gametes increases with structural chromosome aberrations with carriers of reciprocal and Robertsonian translocations demonstrating a 50 and 15 % frequency of unbalanced gametes, respectively [38]. Finally, patients with Y microdeletions also appear to have a small but significant increased risk for sex chromosome aneuploidies [58, 59].

Therefore, although the majority of embryonic aneuploidy is of maternal origin, the sperm genome has a significant role in the formation of a euploid embryo and thus the development of a healthy offspring. As discussed above, in cases of male factor infertility, there is an increased risk for transmitting an aneuploid paternal genome to the oocyte. IVF centers in the UK were surveyed as to whether they routinely perform sperm aneuploidy screening and if they perceive there to be a genetic risk to offspring conceived by intracytoplasmic sperm injection (ICSI) [60]. The vast majority indicated that while their center rarely performed such screening, there was merit in doing so [60]. This opinion gains some weight given that studies to date suggest that there is little to no evidence that aneuploid sperm are at any disadvantage in fertilizing an oocyte compared to a haploid sperm [47, 48, 56]. A handful of studies have convincingly demonstrated a distinct lack of selection against chromosomally abnormal sperm. These studies provide evidence to suggest that increased sperm aneuploidy translates to increased aneuploidy in embryos [61]. The approximate threefold increase in sperm aneuploidy observed in infertile populations is mirrored by a threefold increase in de novo chromosomal abnormalities in children born after ICSI [62]. In the case of chromosomal translocations, the high percentage of chromosomally unbalanced sperm is shown to translate to a high proportion of chromosomally unbalanced embryos [63]. It is clear that certain individuals have an increased risk of producing high levels of sperm aneuploidy including the following: infertile patients (particularly OAT, non-obstructive azoospermia) and patients with structural and numerical chromosome aberrations [38, 47, 48, 56].

### Sperm DNA fragmentation and its impact in the clinic

The need to identify novel markers that can better discriminate between fertile and infertile men and can assess the genetic integrity has given rise to the field of sperm DNA fragmentation. In recent years, this has been of particular interest since with the advent of assisted reproduction technologies, genetic defects that can be transmitted as natural selection barriers to fertilization are bypassed [30]. The effect of abnormal sperm chromatin on subsequent development will depend on the severity of the damage and the repair capacity of the oocyte.

The etiology of sperm DNA damage which is characterized by single- and double-break strands (SSBs and DSBs) is multifactorial and can be related to intrinsic and extrinsic factors. Intrinsic factors include the following: protamine deficiency, excessive reactive oxygen species (ROS) levels, and abortive apoptosis; extrinsic factors include the following: environmental exposures, chemotherapy, and possibly lifestyle factors [8, 64, 65]. As discussed previously during the chromatin remodeling process in sperm, naturally occurring DNA strand breaks occur to facilitate the replacement of histones by protamines. Once chromatin is repackaged, these breaks are subsequently resealed [11]. Perturbations within this machinery of break and repair can cause altered chromatin compaction and residual breaks in the sperm DNA [11] that can result in measurable DNA fragmentation in the ejaculate.

Sperm DNA damage has also been associated with high levels of ROS detected in the semen of approximately 25 % of infertile men [64]. The susceptibility to ROS damage stems from the presence of unsaturated fatty acids in the plasma membrane, necessary for membrane fluidity which is required in the acrosome reaction during fertilization [66]. The only defence mechanism against ROS is the antioxidant ability of the seminal plasma and the sperm chromatin compactness [11]. However, free radicals can be produced both by defective spermatozoa and semen leukocytes thus inducing sperm damage and conferring male subfertility [11, 64, 66]. The window of time during which DNA damage occurs is still under debate but it most likely occurs during the epididymal maturation as this is the period during which spermatozoa are most exposed to ROS [11].

Abortive apoptosis has been postulated as another theory for DNA damage and has been associated with a form of selective apoptosis that, under normal conditions, regulates the production of abnormal sperm in spermatogenesis and limits the population of germ cells to a number that can be supported by the Sertoli cells [11, 64, 67]. Over-expression of this process could lead to oligo- or azoospermia whereas under-expression could give rise to a high proportion of abnormal sperm, which could impair fertilization [67]. Using a marker for apoptosis (Fas), it was found that less than 10 % of apoptotic sperm exist in normozoospermic men whereas approximately 60 % of oligospermic men have more than 10 % of apoptotic sperm [67]. However, other studies have not found this correlation so the jury for the definitive association of DNA fragmentation and apoptotic biomarkers remains elusive [8, 30].

In terms of extrinsic factors, exposure to environmental pollutants (e.g., pesticides and pollution) has also been associated with DNA damage [65, 68]. Chemotherapy treatment in males of reproductive age has been linked with impaired spermatogenesis, increased sperm aneuploidy levels, and increased DNA fragmentation [69, 70]. For the most part, studies have demonstrated that following chemotherapy, recovery of spermatogenesis and return to baseline aneuploidy levels may occur months to years after treatment has ceased [70]. However, DNA damage induced seems to be more persistent than numerical chromosome defects [8, 64, 69]. The impact of lifestyle factors should not be neglected either, since obesity, smoking, and certain occupations (e.g., welding, baking) have been associated with decreased semen quality and increased levels of DNA damage [30, 67, 71].

The widespread use of ICSI and a desire to improve its success rates have led to the incentive to develop assays to test the genetic integrity of sperm. These assays have been developed in an effort to measure sperm chromatin damage, aid in the diagnosis of male infertility, and provide predictive reproductive outcomes. It is beyond the scope of this chapter to review these tests in detail; however, they can be summarized in three groups: (1) sperm chromatin structural probes (e.g., chromomycin A_3_, sperm chromatin structural assay—SCSA); (2) tests that directly assess DNA fragmentation (e.g., TUNEL, COMET assays); and (3) sperm nuclear matrix assays (e.g., sperm nuclear matrix stability assay, chromatin dispersion test) [8]. Several review articles provide more detailed description of each technique with principles, detection methods, and pros and cons of each approach [8, 11, 72, 73].

In order to evaluate the clinical value of the sperm DNA fragmentation tests, it is important to assess the relationship of these tests with pregnancy outcomes. Detailed meta-analyses of published studies have been previously published and will be discussed briefly [8, 30, 73]. A small number of studies have shown that when DNA fragmentation exceeds 30 % with the SCSA test, it indicates a lower likelihood/close to zero probability for fertilization through natural pregnancies and intrauterine inseminations (IUIs) [8, 74]. In terms of IVF, the cohort of studies is quite heterogeneous (*n* > 20) and the trend seems to indicate that lower IVF pregnancy rates is correlated with increased sperm DNA fragmentation. When IVF followed by ICSI comes to the equation, studies suggest surprisingly that level of DNA damage appears not to significantly impact ICSI pregnancies, but this can be attributed to the careful selection of sperm and embryo following ICSI. This selection process likely abrogates the adverse effects of sperm DNA damage. When the DNA fragmentation index is assessed by SCSA, a predictive threshold of 27 % is required for a successful pregnancy via IVF and/or ICSI. However, other groups have demonstrated that pregnancies can occur after ICSI utilizing sperm with a much high proportion of DNA damage [8]. This underlines the requirement for long-term follow-up studies to evaluate any consequences on pregnancies following ICSI utilizing sperm with high levels of sperm DNA damage. It should be noted that studies have also correlated pregnancy loss after IVF or ICSI with high levels of sperm DNA damage [73]. High levels of DNA fragmentation may be unlikely to directly affect fertilization rates since the embryonic genome starts expression at the 4–8 cell stage. Therefore, sperm samples with high levels of DNA damage could have more clinical ramifications during the later stages of embryonic development (i.e., blastocyst), which may in part explain its association with pregnancy loss [73].

A consensus exists within the community that increased sperm DNA fragmentation is associated with a lower chance of conceiving through natural, IUI, or IVF, but not ICSI methods. Current research suggests that couples who may benefit most from assessment of sperm DNA fragmentation are couples with recurrent miscarriages [73] or unexplained male factor infertility [8]. However, the role and impact of sperm DNA fragmentation on fertilization, embryogenesis, and development remains rudimentary at best due to the relatively small number of studies and heterogeneous findings [8, 30, 73]. More studies are needed to better understand the etiology of sperm DNA fragmentation, its potential association with increased risk of pregnancy loss, and identification of the optimal assays. It is clear that these studies are required to further validate the clinical significance of sperm DNA damage before assessment of sperm DNA fragmentation is a routine test in andrology labs and patient management.

### Chromatin organization and modification—the “missing” links with the clinic?

Previously, we have discussed the competence aspects of sperm “chromatin” either in the form of chromosome constitution (aneuploidy) or DNA damage and the impact that their assessment has in the clinic. Such tests have been developed in an effort to provide patients and clinicians with improved diagnostic methods for infertile patient and to better predict ART outcomes. We can thus postulate that a sperm head with the correct chromosome copy number and absence of DNA damage is a “healthy” gamete with increased potential for the faithful transfer from the paternal genome and epigenetic input to the zygote for the development of healthy offspring. Other markers that can be considered important for nuclear health and normal cellular function are the appropriate spatio-temporal organization of the chromatin (i.e., the position of chromosomes and/or genes within the nucleus) and the epigenetic marks that the chromatin carries into the egg.

Chromosomes occupy distinct non-random positions in most interphase nuclei (termed chromosome territories—CTs) [75–80]. This organization appears to be evolutionary conserved, and two models (“gene density related” and “chromosome size related”) have been used to describe the position of chromosomes in different somatic cell types [81]. Although the functional implication of nuclear organization is still an active topic of discussion and beyond the main scope of this chapter, the feature of the distinct non-random position, the evolutionary conservation, and the changes in the patterns of organization observed in certain disease conditions (e.g., laminopathies, Hutchinson-Gilford progeria, breast cancer) [82] highlights the importance for the maintenance of stable architecture for proper cellular function [83].

Any perturbation in nuclear architecture could induce change in the local gene environment and availability to transcription factors leading to possible mis-regulation or failure to take part in transcription [84]. It has been hypothesized that chromatin organization may be crucial for spatial chromatin differentiation, modifications of the epigenome transmitted to the embryo, and normal embryogenesis which may have evolved with other mammalian regulatory systems including genomic imprinting and X inactivation [85, 86]. Thus, the study of nuclear organization in the male gamete could have important ramifications for early embryogenesis when we take into account the unique features of the haploid sperm DNA packaging. Although we have a good understanding of the different chromatin packaging in sperm compared to other cell types, our understanding of the organization of chromatin in spermatogenesis is poor [87].

The vast majority of studies published in this area have been focused solely on the organization of the mature haploid, protamine packaged sperm cell [14, 16, 88–96]. Data emerging from these studies suggest that organization of the human sperm nucleus is different than that of the somatic cells. Chromosomes appear to be clustered via their centromeres to form a chromo-center in the interior of the nucleus, while the telomeres are preferentially located toward the nuclear periphery where they form dimers and tetramers [14–17, 95]. The chromo-center appears to be formed by pericentric heterochromatin from different chromosomes that have a tendency to aggregate [15, 92]. A similar spatial organization has been shown to be evolutionarily conserved in other mammals (mouse [89, 97], bovine, pig, horse, and rat) [95]. The organization of CTs or specific chromosomal regions has been addressed both radially (i.e., location in relation to the nuclear interior to the periphery) and in some studies longitudinally (i.e., distribution in relation to the sperm head and tail) [94]. The hypothesized functional implication of the longitudinal and radial distribution of chromosomes in human sperm is thought to be related to the ordered exodus after fertilization. Thus, the order that the maternal cytoplasmic environment encounters the paternal genome [98] and potentially remodels and repairs DNA damage on the early exiting chromosomes may be important compared to the late exiting regions of the genome. It should also be emphasized that the positions of the sex chromosomes relative to the acrosome are similar in sperm of all mammals (but not birds), implicating a functional significance [86].

Assuming a functional significance for the non-random organization of chromosomes in human sperm with its possible impact in fertilization, it is reasonable to suggest that altered nuclear organization could be a measurable phenotype in the sperm of infertile men [91] and could provide an additional explanation for idiopathic infertility. Early evidence for a clinical impact of chromosome organization in sperm came from observations that sperm used in ICSI which had not undergone the acrosome reaction, showed impaired decondensation of apically located chromatin [16]. Furthermore, this delay in decondensation was observed to hinder the progression of the first mitotic division of the zygote and has provided indirect evidence to potentially explain the increase in sex chromosome aneuploidies observed in offspring after ICSI. This provides a tentative link with the more firmly established clinical impact of sperm aneuploidy and DNA fragmentation in nuclear health. It has been proposed that some infertile men may have a different category of sperm chromatin abnormality related to atypical packing of CTs in sperm, aberrant positioning of chromosomes, or disturbed telomere-centromere interactions [98]. Currently, the proposed relationship between male infertility and altered nuclear organization remains hypothetical, with direct evidence to prove or disprove this link being somewhat lacking. Indeed, only a handful of studies have tried to address this link [88, 92, 99–102]. Currently, we are investigating the 2D and 3D nuclear organization (both radially and longitudinally) of chromosomes within human sperm [94]. Additionally, we are investigating sub-chromosomal structures (telomeres, centromeres), imprinted genes, and important developmental genes that have been shown to retain histones and unique methylation patterns in the protamine packaged sperm. It is clear that the sperm cell can no longer be considered a vessel for delivering a silent genome but is rather an epigenetically poised cell that is crucial for fertilization and embryogenesis.

### Evidence for an epigenetic signature in sperm chromatin that could affect embryogenesis

As indicated above, differential methylation of sperm DNA and the deregulated presence of nucleosomes on embryologically important gene loci appear to correspond with an infertile phenotype [25, 26]. These data suggest the possibility that in addition to the genome itself, sperm chromatin carries an epigenetic ‘signature’ to the egg potentially borne on modified histones. Histone modifications are numerous and beyond the scope of this review [2], but because of the known association of lysine (K)-modified H3 with expression permissive (H3K36me3) and restrictive (H3K27me3) chromatin domains, reports on differential lysine methylation in sperm chromatin are particularly intriguing [7, 25]. These studies follow a raft of earlier reports demonstrating histones in mature sperm, the earliest of which was with CENPA in bull sperm [103] and reviewed in [2, 27]. These reports include microscopic evidence for the transmission and survival to syngamy of paternal nucleosomes. Moreover, biochemical evidence for the transmission and retention of structurally unusual (smaller) sperm nucleosomes containing sperm-specific histone variants (H2AL1 and H2AL2) has also been put forward [104] and potentially confirmed by proteomic analysis [105]. Sperm histones may play an essential role in promoting normal embryogenesis although evidence to date suggests that neither sperm-specific H2AL isoforms, nor any other histone modification persists for long following fertilization [106, 107]. Moreover, as the successful generation of (murine) gynogenic parthenotes following alteration of the *H19/Igf2* imprinted locus demonstrates [108], the paternal genome is actually dispensable altogether with respect to embryonic development. It is more likely, therefore, that an epigenetic signature borne on sperm nucleosomes performs more sperm-centric functions that may nevertheless be prone to deregulation in infertile men, perhaps by aberrant histone deposition as reported elsewhere [25, 109]. In this regard, the essential role of a testis-specific form of the double bromodomain containing BET family, BRDT in facilitating chromatin reorganization during spermiogenesis should be considered, particularly in view of a potential role for this factor in infertility [110, 111]. BRDT is involved in both the formation of the sperm chromo-centre and in the regulation of translational control of stored mRNPs, both vital functions for fertility. On the other hand, the case for some form of histone-based paternal epigenetic contribution is supported by reports that differentially condensed blocks of chromatin containing developmentally important gene sequences analogous to the nucleosome-enriched regions of mammalian sperm chromatin. These are also found in the zebra fish, which does not use protamine to repackage its genome [112]. Furthermore, two other reports have shown that the DNA methylome of the zebra fish egg is fully reprogrammed to resemble the incoming sperm methylome shortly after fertilization [113, 114]. Interestingly, a link between gamete/embryo DNA methylation dynamics and a post-fertilization function for (human) sperm histones was reported earlier in a study showing that DNA methylation-free regions in the early embryo correspond with nucleosome-rich regions in sperm chromatin [115]. These studies and the findings from Hammoud et al. [7] provide supportive evidence of a role for nucleosomal, probably euchromatic regions of the incoming paternal genome with subsequent DNA methylation patterns in the early embryo. However, as the example of gynogenic parthenotes strongly suggests, the sperm’s epigenetic influence is not a prerequisite for subsequent and successful embryonic development.

An alternative possibility is that modified sperm histones introduced into the oocyte on fertilization provide an essential role in facilitating the sperm’s ‘acceptance’ by the egg as a complementary agent. Such a hypothesis is not so outlandish when considering the risk the sperm poses to the oocyte as an invasive cell. Ideas of ‘confrontation,’ ‘recognition,’ and ‘consolidation’ have already been put forward in relation to the potential introduction and management of potentially harmful parasitic mobile elements [116] and also ‘tolerance’ of sex-skewing bacterial endosymbionts such as *Wolbachia* [117, 118]. Such a hypothesis is fully compatible with roles for sperm histones, modified or not, and of course, paternal DNA methylation in male fertility without over stating their importance in the support of embryogenesis per se. The apparent preferential localization of sperm histones to the exome, confirmed in independent studies [7, 18, 119], is likely part of the matching process required for successful syngamy (a particularly attractive notion assuming nucleosomal stretches of sperm chromatin have more immediate access to maternal factors at fertilization than protamine-bound regions). This adds further importance to the potential role of nuclear organization as this may function as an additional layer of epigenetic regulation. Such proposed studies will ultimately identify the spatio-temporal localization of targeted genes throughout spermatogenesis and identify whether nuclear organization is perturbed in infertile men. This field remains an active area of research with possible ramifications for improved screening (in combination with standard tests), diagnosis, and predictions of ART treatment efficacy.

## Conclusions

It is self-evident that the paternal genome is critical for the promotion of normal fertilization and embryogenesis and with infertility affecting approximately one in six couples of the western world and male factor contributing to around 50 % of cases, there is an unequivocal need for further research into the male gamete. Understanding the role(s) played by the sperm’s unique and specialized chromatin structure in conferring a fertile phenotype is also preferable, and the advent of ART makes the evaluation and impact of sperm chromatin structure all the more important. The ultimate goal is the development of rapid reliable tests that can assess the genomic integrity of sperm to be used in ART and to identify novel aspects of chromatin integrity (e.g., genome organization) that may play a crucial role in fertilization and early embryogenesis. The development of such tests outlined in this chapter aims to further our understanding of paternal contribution and requirements for normal fertilization and embryogenesis. The goal is to create an “arsenal” of analytical tools to better diagnose and assess the genetic integrity of the paternal genome to facilitate the transfer of the single euploid embryo. Undoubtedly, the development of reliable tools to assess the integrity of the paternal genome will assist andrologists, embryologists, clinicians, and couples undertaking ART to allow more informed decisions to be taken regarding their reproductive choices.
